# Synergy between Ni and Co Nanoparticles Supported on Carbon in Guaiacol Conversion

**DOI:** 10.3390/nano10112199

**Published:** 2020-11-04

**Authors:** Elodie Blanco, Ana Belen Dongil, Néstor Escalona

**Affiliations:** 1 Departamento de Ingeniería Química y Bioprocesos, Pontificia Universidad Católica de Chile, Santiago 7820436, Chile; neescalona@ing.puc.cl; 2 ANID–Millennium Science Initiative Program—Millennium Nuclei on Catalytic Process towards Sustainable Chemistry (CSC), Santiago 7820436, Chile; 3 Instituto de Catálisis y Petroleoquímica, CSIC, Cantoblanco, 28049 Madrid, Spain; 4 Departamento de Química Física, Facultad de Química y de Farmacia, Pontificia Universidad Católica de Chile, Santiago 7820436, Chile; 5 Unidad de Desarrollo Tecnológico, Universidad de Concepción, Coronel 4191996, Chile

**Keywords:** nickel, cobalt, bimetallic, hydrodeoxygenation

## Abstract

Nickel-cobalt bimetallic catalysts supported on high surface area graphite with different Ni:Co ratios (3:1, 2:1 and 1:1) and the monometallic Ni and Co were prepared by wetness impregnation method. The catalysts were tested in hydrodeoxygenation (HDO) of guaiacol in the liquid phase at 50 bar of H_2_ and 300 °C. The materials were characterized by N_2_ adsorption–desorption, XRD, TEM/STEM, H_2_-TPR, and CO-chemisorption to assess their properties and correlate them with the catalytic results. The activity was higher on the bimetallic catalysts and followed the trend NiCo_2:1_/G ∼ NiCo_3:1_/G > NiCo_1:1_/G > Co/G > Ni/G. Also, selectivity results showed that Ni was more active in the hydrogenation favoring cyclohexanol production from phenol, while this was inhibited on the Co-containing catalysts. Hence, the results showed that synergy was created between Ni and Co and that their interaction, properties, and catalytic performance depend on the metals’ ratio.

## 1. Introduction

Cyclohexanol and phenol are starting materials for producing fuels and plastics such as nylon. These compounds are currently produced mainly by catalytic oxidation of cyclohexane obtained from fossil sources. An alternative to this process is the hydrogenation of biomass-derived compounds, such as those obtained from lignocellulosic wastes. This kind of waste is mainly polymers composed of different monomers, such as coumaryl, coniferyl, and sinapyl, and can be converted into several phenolic compounds through different processes [1,2,3,4,5]. These phenol compounds possess at least one methoxy group that needs to be removed before hydrogenation. For that purpose, guaiacol is one of the most studied model compounds, since it has one hydroxyl and one methoxy group. Hydrodeoxygenation of guaiacol has been extensively studied [4,5], and several catalytic systems have been investigated. Noble metal has been shown to be highly active and led mainly to fully hydrogenated compounds [6,7,8]. Sulfides, phosphides, nitrides, and carbides have proved to be an efficient catalyst for the obtention of aromatics [9,10,11,12,13,14,15]. Non-noble metal has attracted the attention of the obtention of cyclohexanol, especially nickel-based catalysts [16,17,18]. Cobalt-based catalysts have been studied less, but have been found to be active for HDO reactions [19,20,21,22].

Even though nickel-based catalysts were quite active for the obtention of cyclohexanol, in HDO conditions, catalysts suffer from deactivation [23,24,25,26]. Deactivation of the HDO catalysts generally occurs by coking, metal leaching, or sintering [27,28]. To prevent the sintering of the particle, one of the solutions is the use of bimetallic systems [29].

Bimetallic catalysts can improve the stability of the catalyst, and enhance the activity, selectivity toward one product, or create a synergistic/bifunctional effect that would lead to a new reaction pathway. For instance, the addition of Cu to Ni has been shown to diminish the Ni particle size, leading to a decreased hydrogenolysis and deoxygenation reaction [30]. In contrast, the addition of Fe to Ni has shown that over the alloy formed, the C–O cleavage was energetically easier than the monometallic Ni [31,32]. In NiRu bimetallic systems, selectivity toward phenol and the corresponding rate was enhanced compared to monometallic systems [33]. Finally, bimetallic Ni–Co has also been investigated over NiCo/HZSM-5 catalyst, Co was able to strongly stabilized the active Ni sites [34], while over NiCo/Al-MCM-41, the addition of Co could result in better dispersion of the NiO particle leading to a higher HDO activity [35]. On the other hand, in the aqueous phase, bimetallic NiCo supported over Al_2_O_3_, and ZSM-5 has shown that the addition of Co to Ni improved not only the metal dispersion, but also the reducibility and acid sites, enhancing HDO activity [36].

All the NiCo bimetallic catalysts studied involve acidic support, and the synergistic effect between the metal and the support has been discussed and proposed to be helpful for guaiacol HDO. To remove the effect of acid-base properties provided by the support and get some insight into bimetallic systems, the present work aimed to study the effect of the addition of Co to Ni catalysts on inert supports like carbon materials. High surface area graphite was used as support. Moreover, inert support is more stable under reaction conditions since more acidic supports are more prone to react with coke precursors [37,38].

## 2. Materials and Methods 

### 2.1. Catalyst Preparation

Catalysts were prepared by wet impregnation of the support high surface area graphite (HSAG; Timrex 400, supplied by TIMCAL, Bodio, Switzerland) using the corresponding amount of the precursors’ Ni(NO_3_)_2_ (99.9%, Sigma-Aldrich, Merck Group, Darmstadt, Germany) and Co(NO_3_)_2_·6H_2_O (99.9%, Sigma-Aldrich) dissolved in acetone. The metal loading was 15% wt. Ni, 15% wt. of Co in the monometallic catalysts and 15% wt. total metal loading in the bimetallic catalysts, with Ni/Co molar ratio of 3:1, 2:1, and 1:1. The materials were treated at 350 °C for 5 h under flowing N_2_ (50 mL min^−1^) using a heating ramp of 1 °C min^−1^. The samples were previously reduced under H_2_ (50 mL min^−1^) at 300 °C for 4 h and then passivated under a mixture of 5% O_2_/N_2_ for 1 h at low temperature (by using a mixture of liquid nitrogen and isopropylic alcohol). According to this procedure, five catalysts were prepared, which were labeled as Ni/G, NiCo_1:1_/G, NiCo_2:1_/G, NiCo_3:1_/G, and Co/G.

### 2.2. Catalyst Characterization

Textural properties were obtained from the adsorption–desorption isotherm of N_2_ at −196 °C using a 3-Flex instrument (Micromeritics, Norcross, GA, USA). The samples were previously degassed at 300 °C for 4 h under vacuum using a SmartVacPrep instrument (Micromeritics, Norcross, GA, USA). The surface area was determined from the adsorption branch in the range 0.05 ≤ p/p^0^ ≤ 0.25 using the Brunauer–Emmett–Teller (BET) theory [39]. Total pore volume was defined as the single-point pore volume at p/p^0^ = 0.99. 

The crystalline phases present in these samples were determined from the X-ray diffraction patterns (XRD). The diffractograms were recorded on a Polycrystal X’Pert ProPANalytical (EA Almelo, The Netherlands) apparatus using Ni-filtered Cu Kα radiation (λ = 0.15406 nm) and a graphite monochromator. For each sample, Bragg’s angles between 4° and 100° were scanned at a rate of 0.04°. 

TEM was used to obtain information on the particle size and size distribution of metal NPs on the different supports. TEM images were obtained with a JEOL JEM-2100F microscope (JEOL, Tokyo, Japan) at 200 kV. The samples were ground and ultrasonically suspended in ethanol before deposition on a carbon-coated copper grid. The mean diameter d of Ni or Co particles was calculated based on a minimum of 300 particles by using the equation:

(1) $$d=\sum n_{i\;}d_{i}^{3}/\sum n_{i\;}d_{i}^{2}$$

where n_i_ is the number of particles with diameter d_i_.

Temperature programmed reduction (TPR) of fresh catalysts was performed using the Micromeritics 3Flex instrument coupled with a mass spectrometer (Cirrus 2, MKS Spectra Product, Andover, MA, USA). Typically, 15–30 mg of the catalyst in a quartz reactor was heated gradually at 10 °C min^−1^ until 1050 °C in a mixture of 5% H_2_/Ar (100 mL min^−1^). A cold trap (liquid nitrogen with isopropanol) was placed between the reactor and the thermal conductivity detector (TCD) to remove water formed during reduction. During the reduction, the products formed were also monitored in parallel by a mass spectrometer and fragments m/z of 15, 28, 44, corresponding to CH_4_, CO, and CO_2_, were recorded. The H_2_O signal (m/z = 18) was also monitored to ensure that the complete removal of water occurred. TCD signal was calibrated using a standard material, AgO, provided by Micromeritics to quantify the H_2_ consumption during the TPR.

Chemisorption uptakes of CO were measured at 35 °C using a Micromeritics 3-Flex instrument. Typically 30–50 mg of passivated catalyst was first degassed at 110 °C (10 °C min^−1^) for 30 min and then reduced at 300 °C at 1 h. 

The calculation of the average crystallite size of a spherical crystal d was carried out according to Equation (2), wherein S_m_ is the metal surface area calculated from Equation (3) in m^2^ g^–1^ and ρ_m_ the metal density in g m^–3^ (8.9 g cm^−3^ for Ni and Co):

d = 6/(S_m_.ρ_m_) (2)

S_m_ = Fs.n_m._N_A._A_CO_ (3)

where Fs is the stoichiometry factor between CO and the metal, n_m_ the number of mol of CO adsorbed, N_A_ Avogadro’s number, and A_CO_ the cross-sectional area of CO (0.125 nm^2^) 

### 2.3. Catalyst Activity Measurement

Catalysts were evaluated in a 100 mL stirred-batch autoclave set-up (Parr Model 4590, Moline, IL, USA) at 300 °C, stirred at 645 rpm, and under a hydrogen pressure of 5 MPa for 4 h. Then, 0.200 g of catalysts were added to a mixture of 2 mL of guaiacol (reagent grade from Sigma-Aldrich, Merck Group, Darmstadt, Germany), 700 μL of hexadecane (reagent grade from Merck Group, Darmstadt, Germany) as an internal standard, and 75 mL of dodecane as a solvent (reagent grade from Sigma-Aldrich). First, the reactor was purged with a nitrogen flow for 10 min with a (100 mL min^−1^) prior heating. Once the reaction temperature was reached, a first sample was collected, with no previous addition of H_2_ to ensure no guaiacol conversion. The beginning of the reaction was assumed to be when the H_2_ was introduced into the reactor. During the reaction, hydrogen was added to keep the pressure constant. Liquid samples were periodically collected during the reaction and quantified by a Shimadzu (GC-2010 Plus, Kyoto, Japan) gas chromatograph equipped with an Elite-1 column (Perkin Elmer, 30 m × 0.32 mm, film thickness of 0.25 μm). Under such conditions, the reaction rates were not limited by internal or external mass transfer [40]. Conversion (4) and product selectivity (5) were defined according to the following formula:

(4) $$\mathrm{Conversion}\;\left(\%\right)=\frac{n_{\mathrm{GUA}}^{0}-n_{\mathrm{GUA}}}{n_{\mathrm{GUA}}^{0}}\;$$

(5) $$\mathrm{Selectivity}\;\left(\%\right)=\frac{n_{\mathrm{product}\;i}}{\sum n_{\mathrm{products}}}.\;100$$

where n^0^_GUA_ and n_GUA_ correspond, respectively, to the molar quantity measured at t = 0 and t, respectively, and n_product_ *i* is the molar quantity of a product *i*

The initial reaction rate r_0_ (mol g^−1^ s^−1^) was calculated from the initial slope (b) of the conversion vs. time plot (s^−1^) according to the Formula (6):

(6) $$r_{0}=\frac{b\times n_{\mathrm{GUA}}^{0}}{m}$$

where m (g) is the mass of the catalyst. 

Mass balance was about 90% and was determined by comparing the conversion calculated from guaiacol disappearance to that from product formation. 

The turnover frequency (TOF, h^−1^) was defined as the number of mol of guaiacol converted per hour and per surface metallic site of the catalyst. The active sites were considered the total number of metal atoms on the surface, M_s_, which was calculated according to Equation (7) for each metal as a function of the metal atoms, M_t_, and the metal dispersion, D. Where the metal dispersion was calculated by Equation (8):

(7) $$Ms_{\;}\;={\;M}_{t}\cdot D^{\;}$$

(8) $$D\;=\;(6\cdot N_{s}\cdot M_{w})/(\rho \cdot N_{A}\cdot d)$$

where ρ is the density of the metal (8.9 g/cm^3^ for Ni and Co); N_s_ is the number of atoms at the surface per unit area, which has been considered as 1.54 × 10^19^ m^−2^ for Co and Ni; M_w_ is the atomic weight of the element; and N_A_ is Avogadro’s number, and the average particle size is determined by TEM, considering that the particles are spherical. 

## 3. Results

### 3.1. Catalyst Characterization

**Figure 1:**
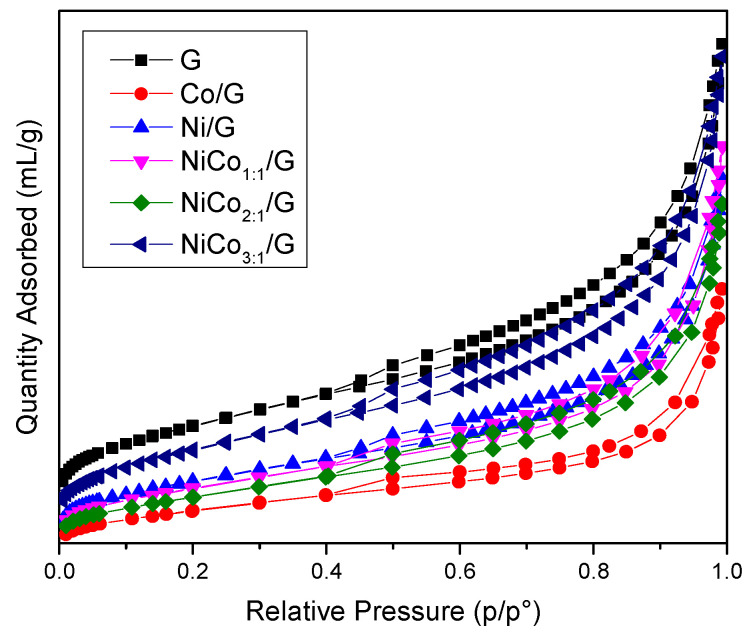
N_2_-sorption isotherm obtained for the support and the prepared catalysts.

Textural properties were measured by N_2_-physisorption, the isotherms obtained for the catalysts and support are presented in Figure 1, and the data are summarized in Table 1.

The figure shows that all the materials exhibited a type IV isotherm with an H4 hysteresis loop corresponding to mesoporous materials with slit-type pores. 

The surface area obtained for the catalysts was systematically lower than the support alone, which was expected as the catalysts were composed of 85% wt of support. However, if the metal was well-dispersed on the support, the surface area should have been around 360 m^2^ g^−1^. Such surface was not reached in any catalyst and indicates that the structure’s changes have occurred, probably due to partial pore-blocking promoted by the metal. This effect was more pronounced in the case of the Co/G catalyst, while in the case of the Ni/G, NiCo_1:1_/G, and NiCo_2:1_/G, a similar external surface area decrease was observed. 

**Figure 2:**
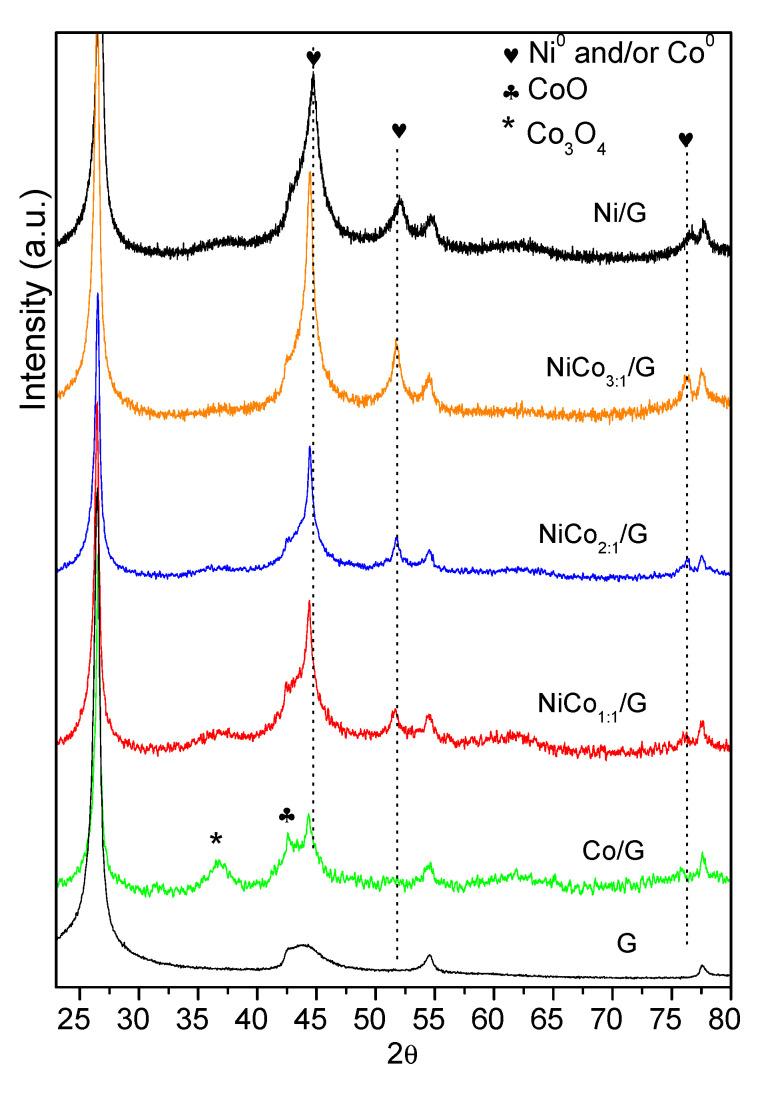
Diffractogram obtained for the support and the prepared catalysts.

The XRD diffraction patterns of the catalysts are shown in Figure 2. Besides the characteristic diffraction peaks of graphite at 26.1°, 43.3° and 54.0° corresponding to the hkl (002) (100) and (004), diffractions ascribed to the metal could be observed. The monometallic catalyst Ni displayed the characteristic Ni^0^ peaks at 44.4°, which overlapped with that of the (100) diffraction of graphite at 51.8° and 76.4°, corresponding to the diffractions in the (200) and (220) crystalline planes of the fcc phase, (JCPDS 04-0850). Even though diffraction of the (200) plane of NiO phase may have overlapped with that of graphite at 43.3°, the absence of the diffraction peaks at 37° and 64° from NiO seems to indicate that nickel oxide was mostly absent. 

On the other hand, monometallic Co catalyst displayed the diffraction peaks of Co^0^ at 45.9° (111), which overlapped with that of graphite at 43.3° and that at 2θ of 53.5° due to the plane (200) was insinuated (JCPDS 88-2325), along with those of the Co_3_O_4_ at 36.6° (311) (JCPDS 42-1467) and CoO at 42.7° (200) (JCPDS 78-0431). Although the samples were shortly exposed to air (see Section 2.1 and Section 2.2), the presence of the Co_3_O_4_ phase could be indicative of an incomplete reduction during the activation of the catalysts.

As far as bimetallic samples are concerned, Ni and Co have very similar molecular weight and fcc crystal lattice, making the comparison of bimetallic diffractograms challenging. 

The bimetallic Ni–Co catalysts showed three major diffraction peaks at around 44.3–44.7°, 51.6–52.1°, and 76.1–76.5°, which corresponded to the (111), (200), and (220) crystalline planes of either pure metals or Ni–Co alloy (PDF [01-074-5694) [41]. The maximum of each diffraction peak shifted to lower diffraction angles upon increasing cobalt content as the dotted lines evidenced, which could be due to the partial replacement of Ni to Co, leading to the formation of Ni–Co alloy or the interaction between Ni and Co [42]. Since the diffraction peaks of pure Ni and Co overlap with those of the Ni–Co, the presence of individual entities Ni or Co cannot be ruled out. Moreover, in the bimetallic samples, no clear diffractions could be observed due to nickel or cobalt oxides. 

**Figure 3:**
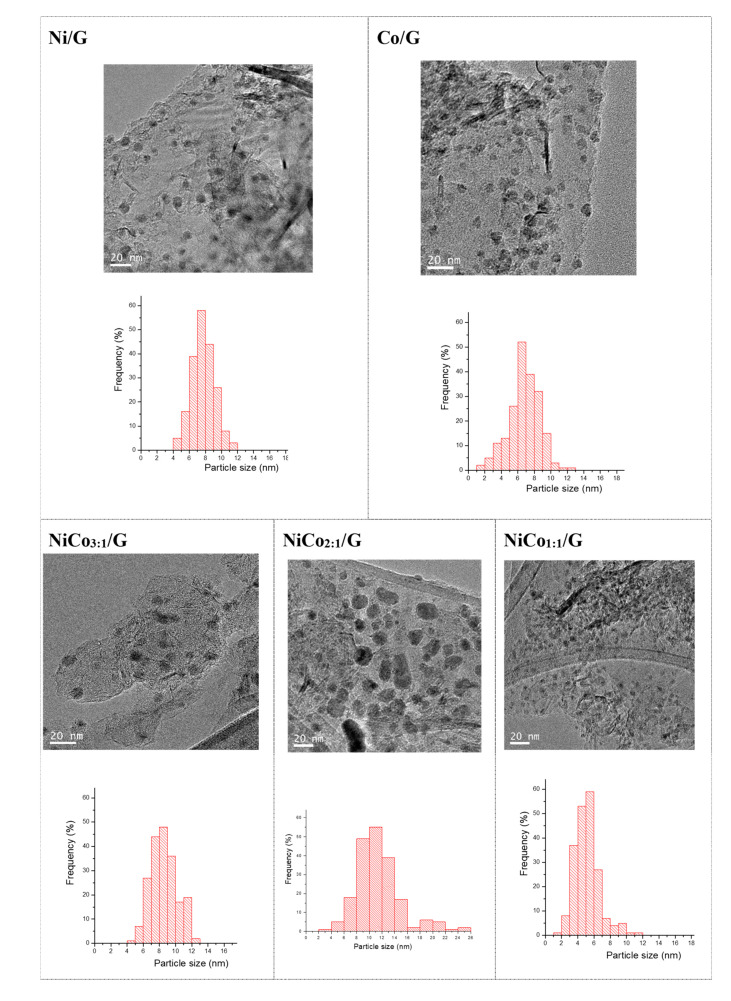
TEM images and histograms of the catalysts.

Figure 3 displays the TEM images obtained for each catalyst and the corresponding average particle size distribution. The figure shows that in the case of the monometallic catalysts, similar particle size distribution was obtained. The average was centered at 7 nm in the case of Ni and 8 nm for Co. 

In the bimetallic catalysts, the average particle size distribution changed depending on the Ni:Co ratio. The addition of Co to Ni resulted in an increase of the average particle size compared to pure Ni catalyst for the ratios of 3:1 and 2:1. Interestingly, in the NiCo_1:1_/G catalyst, the smallest average particle size was obtained (ca. 5 ± 1 nm). 

CO chemisorption was carried out to evaluate the metallic particle size of each catalyst. The stoichiometry factor considered was one with a spherical morphology as observed by microscopy. Results are presented in Table 1 and are in good agreement with TEM results for Ni but differ strongly with the results obtained for Co, e.g., 25 nm by CO chemisorption vs. 8 nm by TEM. An explanation could be the different sensitivity of Co to CO chemisorption compared to Ni. For instance, Bartholomew et al. have reported that the adsorption mode of CO on Co varied with the particle size [43], so that the stoichiometric factor changes, leading to an underestimation of the particle size in the case of Co. In bimetallic catalysts, the value was also underestimated and did not follow the same trend observed by TEM. This suggests that, for these catalysts, CO chemisorption is not a suitable method to estimate the particle size as CO chemisorption stoichiometry can be affected by the particle size.

Nonetheless, CO uptake presented in Table 1 can give some insight into the metallic-like character of the bimetallic catalysts. For instance, if there were no interactions between Ni and Co, CO uptake should continually decrease by increasing the amount of Co, since Co has been shown to weakly chemisorb CO compared to Ni. However, in the present case, CO uptake obtained for all the bimetallic catalysts were quite similar, and the uptake was close to the one measured for Co. This behavior suggests that even a small amount of Co has inhibited the interaction between Ni and CO, suggesting that Ni may have shared its electrons, leading to a lower metal-like character. 

**Figure 4:**
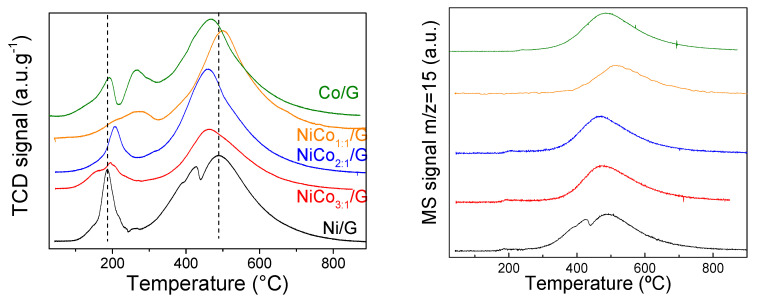
TCD profile (**left**) and MS signal of the m/z = 15 fragment (**right**) obtained for all the catalysts during the temperature programmed reduction (TPR).

TPR analyses of the catalysts followed by TCD and coupled with mass spectrometry were carried out to investigate those interactions further, and the results are presented in Figure 4. 

The TCD signal displayed, in all the cases, two distinct regions: one at low temperature (below 300 °C) and another one at a higher temperature. According to the fragments followed by mass spectroscopy, the evolution of m/z 28 or 44 was negligent in the whole experiment, and no other compounds were found in the low-temperature range, indicating that the TCD signal in that region can be associated with H_2_ consumption. In the case of the band at a higher temperature, the secondary MS signal of CH_4_ (m/z 15) was detected (Figure 4). This behavior has already been reported in the case of other carbon-based supported nickel and cobalt catalysts and was ascribed to methanation of the support [44,45,46]. The temperature at which the methanation reaction occurs depends on the active species for the same support, and, as observed in Figure 4, it shifted to lower values, ca. 457 °C, for the catalysts with 2:1 and 3:1 ratio and the monometallic Co. This can be related to either particle size, the exposed active phase, or, as previously suggested to the strength of metal–support interaction [47]. 

According to the literature review, bulk NiO can be reduced at 365 °C [45]. By supporting NiO, reduction temperature changes as a function of the interaction with the support, which depends mainly on the particle size and surface chemistry [48]. The TPR of Ni/G showed a single well-resolved peak at 185 °C and a shoulder at around 148 °C, which corresponded to the reduction in unreduced NiO to Ni or the passivation layer. Furthermore, the narrow shape of the band suggests a homogenous average particle size, in agreement with TEM results. The TPR of Co/G presented two broad bands at 192 °C and 265 °C, assigned respectively to the reduction of Co_3_O_4_ to CoO and the reduction of CoO to Co [21]. 

The TPR profile obtained for the bimetallic catalysts was quite different depending on the Ni/Co ratio. In the case of the ratio 3:1, two broad bands at 156 °C and 194 °C were observed. The low-temperature band can be assigned to the reduction of NiO and/or Co_3_O_4_, whose reduction has been favored by the addition of the second metal. A similar shape was observed in the case of the ratio 1:1, but at a higher temperature (ca. 209 °C and 271 °C). This shift can be assigned to the decrease in the particle size, as observed for NiCo_1:1_/G catalyst by TEM. Interestingly for the NiCo_2:1_/G catalyst, the profile obtained was similar to Ni alone but shifted to a higher temperature (ca. 204 °C with a shoulder at 170 °C), and this change cannot be explained by changes in the particle size distribution as the NiCo_2:1_/G catalyst presented the larger particle size. Thus, it is possible that the NiO particles decorated and/or covered the Co particles, leading to a higher reduction temperature due to strong interaction among them. 

**Figure 5:**
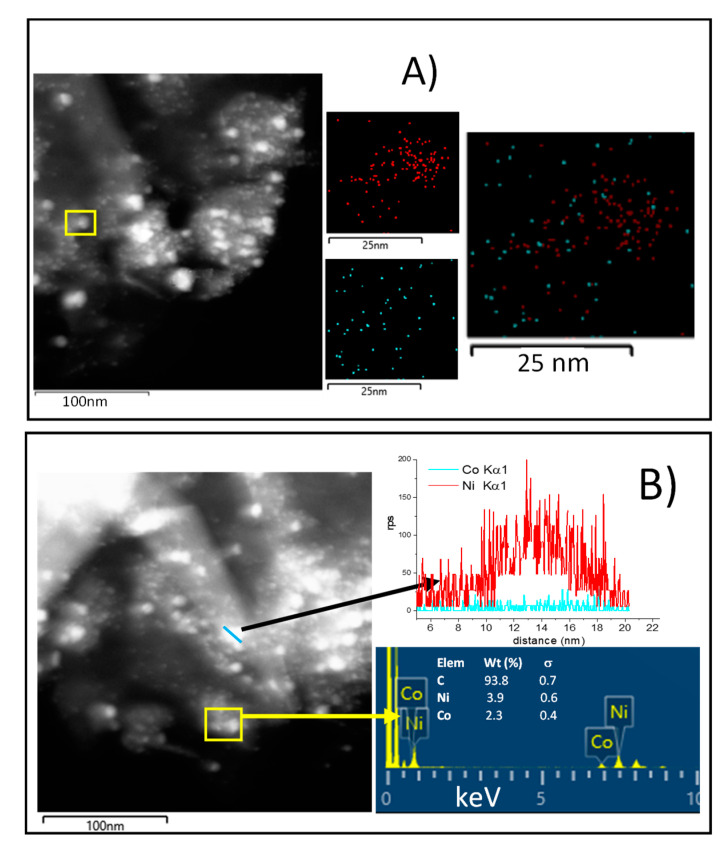
(**A**) Dark-field STEM image of NiCo_1:1_/G with the corresponding EDS elemental maps of the highlighted area showing the chemical distribution of Ni (red), Co (blue); (**B**) EDS line-scanning of NiCo_2:1_/G Ni (red), Co (blue).

To further investigate the samples, STEM images with EDS analysis were carried out on NiCo_1:1_/G and NiCo_2:1_/G catalysts, and the results are provided in Figure 5A,B, respectively. 

EDS mapping of the NiCo_1:1_/G shows that this catalyst exhibited a homogenous distribution of Co in the particle, while Ni particles appeared more agglomerated. The superposition of the mapping of the two metals seems to indicate that individual Ni and Co particle were obtained together with some Ni–Co particles. Among the Ni–Co particles obtained, it appears that different stoichiometry could be obtained, some of the Ni–Co particles were stochiometric while others were Co or Ni-rich. This evidenced that there was the formation of alloy and that the alloy obtained could present different stoichiometry.

On the other hand, the results observed for NiCo_2:1_/G (Figure 5B) also indicate that both individual Ni nanoparticles (as shown by the line scanning), along with Ni–Co particles, in a proportion quite close to the theoretical, exist. Those Ni–Co particles could be the results of alloys formation or core@shell. 

To summarize, monometallic Ni and Co/G catalysts presented similar particle size distribution. Ni exhibited a higher sensitivity to CO chemisorption than Co, suggesting a higher metal-like character. 

For the bimetallic catalysts, the particle size distribution depended on the Ni:Co ratio. Particles were larger for the 3:1 and 2:1 ratio, while for the 1:1 ratio, the particles obtained were smaller. On the other hand, XRD, CO chemisorption, and TPR pointed out that there was some interaction between Ni and Co that could result from an alloy formation. STEM-EDS analysis suggested the obtention of alloy phases with different stoichiometry together with individual monometallic particles. 

### 3.2. Catalytic Properties

The different catalysts were evaluated in the guaiacol conversion at 300 °C, under 50 bar of H_2_. The initial rate of conversion, r_0_, and the TOF calculated are presented in Table 2. TOF was obtained according to the metal surface estimated from particle size by TEM, as the CO chemisorption has been shown to be not reliable for estimating the metal surface.

**Figure 6:**
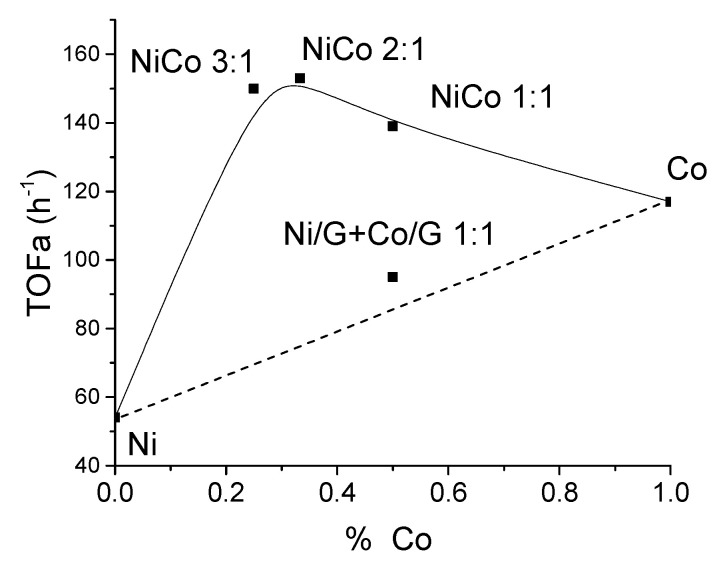
Evolution of the TOF conversion with the Co content in the catalysts.

For the monometallic catalysts, Ni catalyst displayed a lower activity than Co catalyst. This behavior has already been reported in other cases, such as Ni/Co supported over Al-MCM-41 or Zirconium phosphate (ZrP) [22,49]. To better observe the effect of the bimetallic systems compared to the monometallic catalysts, we have represented in Figure 6 the theoretically expected trend (dotted line) if there were no interaction or synergy between the two metals, along with the estimated experimental values (symbols). On the other hand, an experiment with the mechanical mixture of Ni/G and Co/G (1:1) was carried out to evaluate better if there is a synergistic effect. 

This figure shows that the addition of cobalt improved the activity and that every bimetallic catalyst displayed higher activity than the monometallic catalysts. Nonetheless, the values for the bimetallic catalysts were quite similar, which could suggest similar active sites. Interestingly, for the mechanical mixture, the TOF value was much lower than the homolog bimetallic catalysts, and the value obtained was quite close to the expected value (dotted line). 

According to the characterization results, it has been shown that the average particle size of the bimetallic catalysts was larger than the monometallic catalyst, except for the NiCo_1:1_/G, which displayed the smaller particle size. Therefore, the improvement of the activity cannot be associated with better metal dispersion.

Hence, based on the TOF values for the bimetallic catalysts, this behavior can be related to the interaction between Ni and Co as the CO chemisorption, H_2_-TPR, XRD, and microscopy suggested, and it can be inferred that some synergy has emerged between Ni and Co. This synergism could be ascribed to the formation of a new phase (such as an alloy) more active and/or to some electronic transfer between Ni and Co, as already evidenced in other NiCo systems [50].

**Figure 7:**
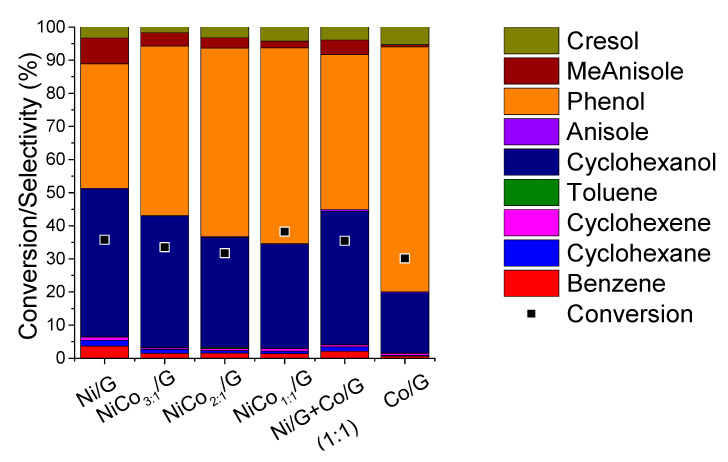
Distribution of the products obtained for each catalyst at 300 °C, 5.0 MPa compared at the same conversion.

**Figure 8:**
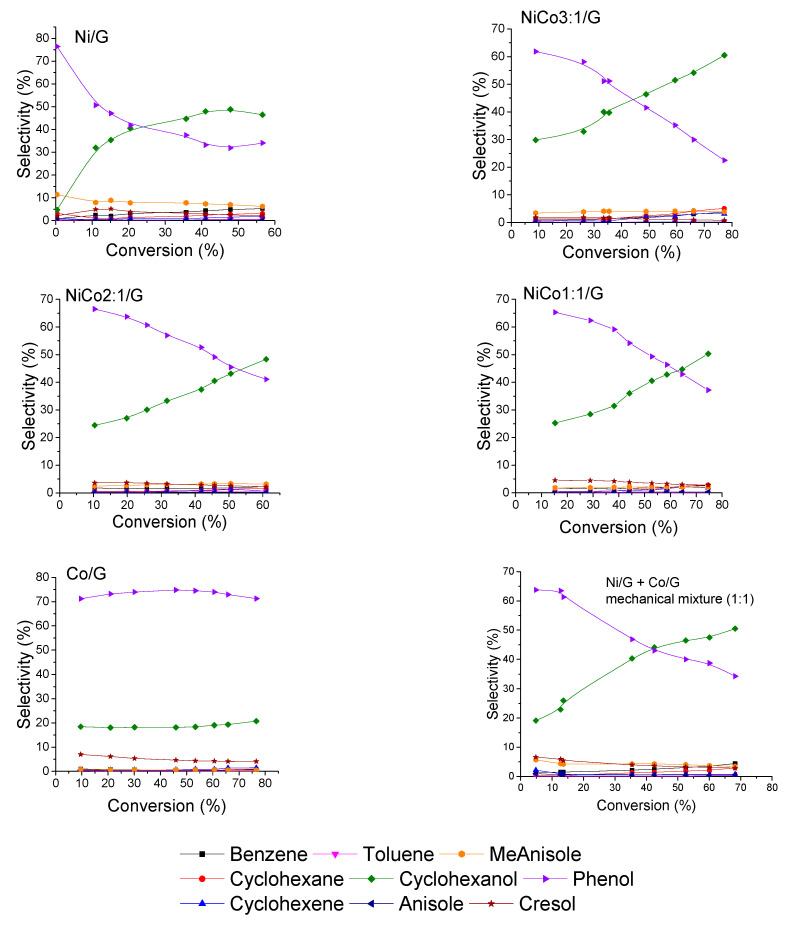
Evolution of the product selectivities with guaiacol conversion for each catalyst at 300 °C, 50 bar.

A comparison of the product distribution at similar conversion, ca. 30%, was presented in Figure 7, while the evolution of the product selectivity with the conversion was plotted in Figure 8. 

Figure 7 shows that for the monometallic catalysts, the distribution obtained was quite different. Phenol and cyclohexanol were the primary product on Ni catalyst, and according to the evolution of the product selectivity with the conversion (Figure 8), the formation of cyclohexanol was obtained from phenol hydrogenation. On the other hand, for Co catalyst phenol was the main product obtained, and based on the evolution of phenol selectivity with conversion, the further hydrogenation to cyclohexanol was inhibited, indicating that Co was less active for hydrogenation than Ni. Those observations are in agreement with the results reported for phenol conversion over Co/HZSM-5 catalyst, where it was observed that phenol conversion was much lower than Ni/HZSM-5 [34]. Nevertheless, in the case of the Ni/HZSM-5, the main product obtained was cyclohexane, while in our case, cyclohexanol was the final product; such difference could be ascribed to the difference in the acidity of the support [18,34,41]. In fact, in the case of bimetallic NiCo-based catalyst, different supports were compared in the conversion of phenol, and the product distribution was able to be adjusted by the support acidity [41]. On the other hand, a study compared catalytic properties of Co and Ni supported over SiO_2_ and reported that over Co, benzene was the main product at complete conversion, while over Ni, cyclohexanol was mainly obtained [51]. 

In the case of the bimetallic catalysts, by increasing the amount of Co, the formation of cyclohexanol was inhibited, leading to higher selectivity in phenol. This suggests that phenol hydrogenation can be limited and is in agreement with the CO-chemisorption results, where the lower metal-like character of the bimetallic catalyst was noticed compared to Ni. Furthermore, the product distribution of the NiCo_1:1_/G catalysts differed from the mechanical mixture. Thus, the difference in product distribution observed for the bimetallic catalyst can be associated with a synergistic effect between Ni and Co. Such synergism could have emerged from the formation of alloys, as observed by STEM that induces some electronic effect (reducing the metal-like character), affecting reactivity. Recently, it was reported over bimetallic Ni–Co/Al_2_O_3_ catalysts that CoO decorates the NiCo alloy particles originating some surface oxygen-vacancies, i.e., defective CoO_x_ species, and this was proposed as the explanation of the synergism between the two metals in the HDO of vanillin [50]. The vacancy would act as an adsorbing site for the oxygen atom of the carbonyl moiety. Similar conclusions were suggested in the case of bimetallic MoO_x_–ReO_x_/SiO_2_ for phenol HDO [52]. The formation of such species, CoO_x_, was favored on the bimetallic catalysts due to the electronic interactions between both.

Thus, the present synergistic effect observed could also be related to the presence of oxygen-vacancies from the superficial CoO_x_ species covering the alloy nanoparticles, which would explain the lower CO uptake observed for the bimetallic catalysts compared to Ni. This synergy seems to be reflected as better adsorption of guaiacol, improving the initial rate of conversion. 

To evaluate the transformation’s path of guaiacol over the different catalysts, the evolution of product selectivity with conversion is presented in Figure 8. The figure shows that guaiacol was first converted into phenol for all the catalysts prepared. Interestingly, no methoxycyclohexanol was detected, while according to the literature review, some Ni-based catalysts gave methoxycyclohexanol as a primary product [30,48,49,53,54]. An explanation could be the different operating condition. Indeed, Lu et al. have studied the effect of the reaction condition in guaiacol conversion for Ni/SiO_2_-TiO_2_ catalyst and have shown that low temperature and high pressure favored the formation of methoxycyclohexanol, while high temperature inhibited such reaction at the benefit of phenol formation [53]. For instance, previous work on Ni supported on several carbon-based supports has demonstrated, under the same reaction conditions to those employed here, the formation of methoxycyclohexanol [30,48,54]. Hence, the absence of methoxycyclohexanol in the catalysts evaluated in the present work cannot be ascribed to the reaction conditions, and the differences could be tentatively explained by the different carbon support used. Thus, it is possible that the carbon nanotubes employed before could promote the transport of H_2_ by spillover, favoring the formation of methoxycyclohexanol, as it has been widely reported back for several catalytic systems [55]. This behavior is less likely for the carbon support used now, which owns more defects, i.e., less conductive character. Therefore, the hydrogenative properties of Ni might be tuned by changing the support. 

Another aspect that can be discussed is the evolution of phenol selectivity with the conversion. In the case of Ni catalyst, at low conversion, e.g., below 10%, the initial rate of phenol transformation to cyclohexanol was much higher than at higher conversion, and for conversion higher than 40%, the hydrogenation seems to have been inhibited. Such behavior could be indicative of deactivation or modification of the active site, inhibiting the hydrogenation reaction of phenol. 

On the other hand, over the bimetallic catalysts, the transformation of phenol to cyclohexanol was carried out with the same rate in the conversion range studied. This suggests that the deactivation and/or modification of the active site can be avoided by the addition of Co. Furthermore, it seems that the formation rate of cyclohexanol decreased with the addition of cobalt, which is consistent with the products obtained by the Co catalysts. 

**Figure 9:**
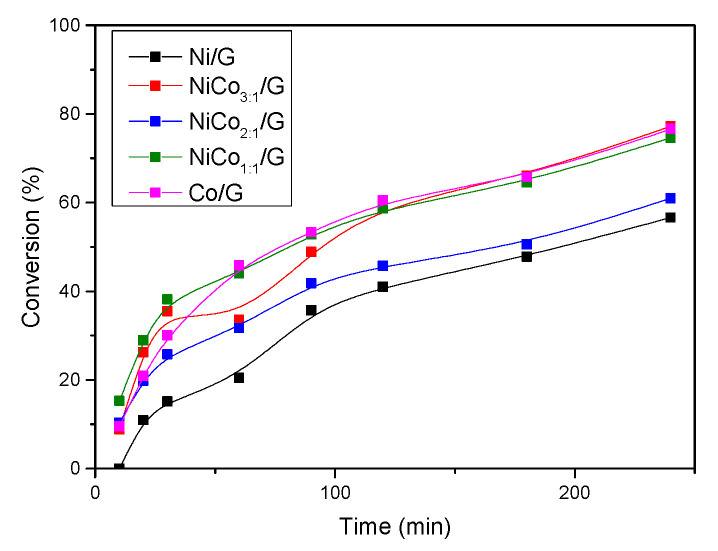
Evolution of guaiacol conversion with time for each catalyst at 300 °C, 50 bar.

To further investigate, the evolution of guaiacol conversion with time is presented in Figure 9. The figure shows that for Ni/G catalyst, the evolution of guaiacol conversion with time was almost linear below 20%, suggesting that the change in the rate of phenol conversion was more likely due to the modification of active sites rather than deactivation. Furthermore, the figure shows that the rate of guaiacol conversion decreased with time for all the catalysts. This could be due to the modification of the active site, particle sintering, or coke deposition. In the case that there are no changes in the active site, this behavior can be ascribed to a change in the reaction order or to other effects like the competition between two reactions.

Since monometallic Ni/G and NiCo_1:1_/G catalysts displayed the smallest particle size, they were characterized after reaction to assess for their stability and compared to Co/G. The spent catalysts were characterized by N_2_-physisorption and compared to the fresh catalyst to evaluate if a surface loss occured due to coke deposition. The results are presented in Table 3 and show that the changes in S_BET_ observed can be assigned to the experimental error. Such results indicate that there was no evidence of deactivation by pore blocking that could have originated from coke formation. 

The particle size estimated using TEM images, in Table 3, indicates that barely any sintering occurred on Ni/G and NiCo_1:1_/G. On the contrary, the monometallic Co catalyst showed a much larger particle size after the reaction compared to the fresh catalyst.

**Figure 10:**
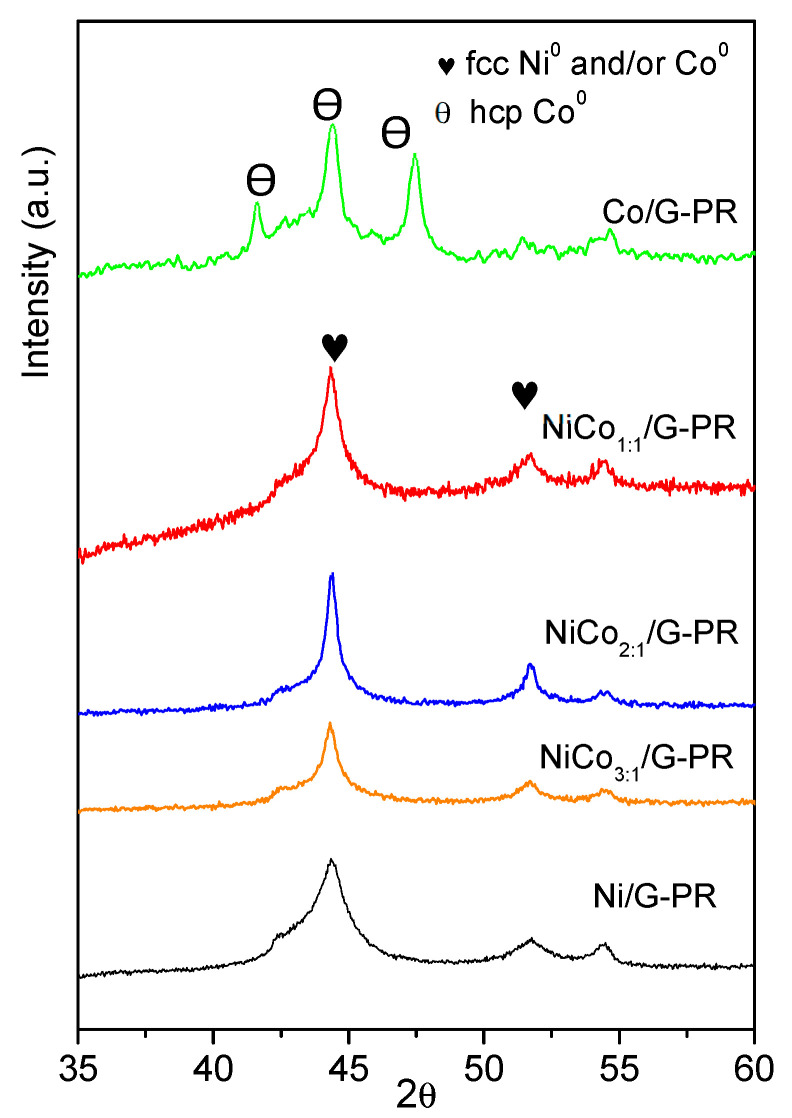
XRD pattern obtained for the spent catalysts.

XRD patterns of the spent catalysts are presented in Figure 10 and show that for the Ni/G spent catalyst, the XRD pattern obtained was similar to the fresh catalyst, suggesting that during the reaction, the Ni^0^ phase was not oxidized to NiO. Moreover, no peaks associated with pure Ni or Co metals were observed on the bimetallic sample, suggesting that the alloy structure was preserved.

Interestingly, in the case of the Co spent catalyst, the diffractogram obtained was quite different to the fresh catalyst. The diffraction peak observed at 2θ of 41.6°, 44.4°, 47.4° could be assigned to the hexagonal hcp-Co^0^ phase or Co_2_C phase (PDF-01-072-1369). 

According to the literature, the fcc-Co phase was obtained under the reduction of the oxide under H2 and was stable above 450 °C, while the formation of hcp-Co has been reported to occur by reducing the oxide under CO+H_2_ mixture [56]. On the other hand, it has been reported that hcp-Co could be obtained by decomposition of Co2C [57]. Finally, the transition between the fcc and hcp phase could have originated from the change in the average particle size [58]. Therefore, at this point, it cannot be ruled out if the transition phase was the result of the carburization of the Co during the reaction or the particle sintering.

An important observation was the absence of the diffraction peak related to the oxide phase (ca. 37°, 43°, and 64° for NiO, 36°, 42°, and 62° for CoO, and 36°, 56°, 60°, and 65° for Co_3_O_4_), indicating that during the reaction no oxidation of the active phase occured.

## 4. Conclusions

The catalytic performance of bimetallic NiCo catalysts supported on a high surface area graphite and that obtained with the monometallic catalysts with the same total metal weight percentage were evaluated. The activity was better for the bimetallic catalysts, and the best catalyst among them was that with a Ni:Co ratio of 2:1, which displayed higher activity compared to Ni/G and Co/G by a factor of 2.9 and 1.3, respectively, while the experiment with a mechanical mixture of Ni/G and Co/G (1:1) displayed a lower activity than the bimetallic catalysts. Moreover, differences in the selectivity were also observed. The product distribution evaluated at the same conversion showed that monometallic Ni was more active on the hydrogenation of phenol to cyclohexanol, these products being the most abundant on the reaction media. However, the addition of Co inhibited the hydrogenation, this being more pronounced for the catalysts with higher Co content, NiCo_2:1_/G and NiCo_1:1_/G.

The results can be explained by the interaction between Ni and Co on the bimetallic samples, which was observed by the characterization performed (TEM, XRD, CO-chemisorption, and TPR). Such interactions might have been generated by the formation of alloys as detected by STEM and XRD. The characterization also showed that the formation of alloy decreased the particle size of Co, and it also prevented the phase transition during the reaction that was observed for monometallic Co. Moreover, the beneficial effect on the particle size was more pronounced on the stability of the nanoparticles since the monometallic spent Co catalyst displayed a much larger particle size than the fresh catalyst. This was not observed on the bimetallic systems, so the formation of bimetallic alloys greatly favored the stability of the catalysts, which is very interesting for industrial applications. 

Hence, according to our results, a synergy was created among Ni and Co, since improved reducibility, smaller particle size, and better activity, selectivity, and stability could be obtained using bimetallic Ni:Co catalysts supported on carbon. 

## Figures and Tables

**Table 1:** Physico-chemical properties of the resulting catalysts.

		G	Ni/G	NiCo_3:1_/G	NiCo_2:1_/G	NiCo_1:1_/G	Co/G
S_BET_ (m^2^ g^−1^)		422	272	289	260	254	194
Vp (mL g^−1^)		0.62	0.46	0.51	0.50	0.50	0.33
CO uptake (µmol g^−1^)		-	311	81	104	123	96
Particle size (nm)	CO-chem.	-	8	31	24	20	25
	TEM	-	7 ± 1	9 ± 1	11 ± 2	5 ± 1	7 ± 1

**Table 2:** Initial rate of guaiacol conversion and the turnover frequency (TOF) calculated for each catalyst.

Catalyst	Ni/G	NiCo_3:1_/G	NiCo_2:1_/G	NiCo_1:1_/G	Ni/G + Co/G (1:1) *	Co/G
r^0^ (10^−6^ mol g^−1^ s^−1^)	8.0	21.6	16.9	23.5	11.5	19.9
TOF (h^−1^)	54	150	153	139	68	117

* Mechanical mixture of Ni/G and Co/G (1:1).

**Table 3:** Comparison of the surface area measure for the fresh and spent catalysts and average particle size of the spent catalysts obtained by TEM.

S_BET_ (m^2^ g^−1^)	Ni/G	NiCo_1:1_/G	Co/G
Fresh	272	254	194
Spent	263	227	207
d_TEM_ (nm)	7	6	>50

## References

[1] Joffres B., Lorentz C., Vidalie M., Laurenti D., Quoineaud A.A., Charon N., Daudin A., Quignard A., Geantet C. (2014). Catalytic hydroconversion of a wheat straw soda lignin: Characterization of the products and the lignin residue. Appl. Catal. B.

[2] Pandey M.P., Kim C.S. (2011). Lignin Depolymerization and Conversion: A Review of Thermochemical Methods. Chem. Eng. Technol..

[3] Ragauskas A.J., Beckham G.T., Biddy M.J., Chandra R., Chen F., Davis M.F., Davison B.H., Dixon R.A., Gilna P., Keller M. (2014). Lignin Valorization: Improving Lignin Processing in the Biorefinery. Science.

[4] Zakzeski J., Bruijnincx P.C.A., Jongerius A.L., Weckhuysen B.M. (2010). The Catalytic Valorization of Lignin for the Production of Renewable Chemicals. Chem. Rev..

[5] Saidi M., Samimi F., Karimipourfard D., Nimmanwudipong T., Gates B.C., Rahimpour M.R. (2014). Upgrading of lignin-derived bio-oils by catalytic hydrodeoxygenation. Energy Environ. Sci..

[6] Boonyasuwat S., Omotoso T., Resasco D.E., Crossley S.P. (2013). Conversion of Guaiacol over Supported Ru Catalysts. Catal. Lett..

[7] Nimmanwudipong T., Aydin C., Lu J., Runnebaum R.C., Brodwater K.C., Browning N.D., Block D.E., Gates B.C. (2012). Selective Hydrodeoxygenation of Guaiacol Catalyzed by Platinum Supported on Magnesium Oxide. Catal. Lett..

[8] Chang J., Danuthai T., Dewiyanti S., Wang C., Borgna A. (2013). Hydrodeoxygenation of Guaiacol over Carbon-Supported Metal Catalysts. ChemCatChem.

[9] Bui V.N., Laurenti D., Afanasiev P., Geantet C. (2011). Hydrodeoxygenation of guaiacol with CoMo catalysts. Part I: Promoting effect of cobalt on HDO selectivity and activity. Appl. Catal. B.

[10] Feitosa L.F., Berhault G., Laurenti D., da Silva V.T. (2019). Effect of the Nature of the Carbon Support on the Guaiacol Hydrodeoxygenation Performance of Nickel Phosphide: Comparison between Carbon Nanotubes and a Mesoporous Carbon Support. Ind. Eng. Chem. Res..

[11] Ghampson I.T., Sepulveda C., Garcia R., Frederick B.G., Wheeler M.C., Escalona N., DeSisto W.J. (2012). Guaiacol transformation over unsupported molybdenum-based nitride catalysts. Appl. Catal. A.

[12] Jongerius A.L., Gosselink R.W., Dijkstra J., Bitter J.H., Bruijnincx P.C.A., Weckhuysen B.M. (2013). Carbon Nanofiber Supported Transition-Metal Carbide Catalysts for the Hydrodeoxygenation of Guaiacol. ChemCatChem.

[13] Blanco E., Dongil A.B., García-Fierro J.L., Escalona N. (2020). Insights in supported rhenium carbide catalysts for hydroconversion of lignin-derived compounds. Appl. Catal. A.

[14] Leiva K., Martinez N., Sepulveda C., García R., Jimenez C.A., Laurenti D., Vrinat M., Geantet C., Fierro J.L.G., Ghampson I.T. (2015). Hydrodeoxygenation of 2-methoxyphenol over different Re active phases supported on SiO_2_ catalysts. Appl. Catal. A.

[15] Blanco E., Aguirre-Abarca D.A., Díaz de León J.N., Escalona N. (2020). Relevant aspects of the conversion of guaiacol as a model compound for bio-oil over supported molybdenum oxycarbide catalysts. New J. Chem..

[16] Alda-Onggar M., Mäki-Arvela P., Aho A., Simakova I.L., Murzin D.Y. (2019). Hydrodeoxygenation of phenolic model compounds over zirconia supported Ir and Ni-catalysts. React. Kinet. Mech. Catal..

[17] Lindfors C., Mäki-Arvela P., Paturi P., Aho A., Eränen K., Hemming J., Peurla M., Kubička D., Simakova I.L., Murzin D.Y. (2019). Hydrodeoxygenation of Isoeugenol over Ni- and Co-Supported Catalysts. ACS Sustain. Chem. Eng..

[18] Barton R.R., Carrier M., Segura C., Fierro J.L.G., Park S., Lamb H.H., Escalona N., Peretti S.W. (2018). Ni/HZSM-5 catalyst preparation by deposition-precipitation. Part 2. Catalytic hydrodeoxygenation reactions of lignin model compounds in organic and aqueous systems. Appl. Catal. A.

[19] Ghampson I.T., Sepúlveda C., Dongil A.B., Pecchi G., García R., Fierro J.L.G., Escalona N. (2016). Phenol hydrodeoxygenation: Effect of support and Re promoter on the reactivity of Co catalysts. Catal. Sci. Technol..

[20] Ranaware V., Verma D., Insyani R., Riaz A., Kim S.M., Kim J. (2019). Highly-efficient and magnetically-separable ZnO/Co@N-CNTs catalyst for hydrodeoxygenation of lignin and its derived species under mild conditions. Green Chem..

[21] Liu X., Jia W., Xu G., Zhang Y., Fu Y. (2017). Selective Hydrodeoxygenation of Lignin-Derived Phenols to Cyclohexanols over Co-Based Catalysts. ACS Sustain. Chem. Eng..

[22] Tran N.T.T., Uemura Y., Ramli A. (2016). Hydrodeoxygenation of Guaiacol over Al-MCM-41 Supported Metal Catalysts: A Comparative Study of Co and Ni. Procedia Eng..

[23] Mortensen P.M., Gardini D., de Carvalho H.W.P., Damsgaard C.D., Grunwaldt J.-D., Jensen P.A., Wagner J.B., Jensen A.D. (2014). Stability and resistance of nickel catalysts for hydrodeoxygenation: Carbon deposition and effects of sulfur, potassium, and chlorine in the feed. Catal. Sci. Technol..

[24] Bykova M.V., Zavarukhin S.G., Trusov L.I., Yakovlev V.A. (2013). Guaiacol hydrodeoxygenation kinetics with catalyst deactivation taken into consideration. Kinet. Catal..

[25] Li Y., Zhang C.S., Liu Y.G., Hou X.X., Zhang R.Q., Tang X.Y. (2015). Coke Deposition on Ni/HZSM-5 in Bio-oil Hydrodeoxygenation Processing. Energy Fuels.

[26] Li Y., Zhang C.S., Liu Y.G., Tang S.S., Chen G.H., Zhang R.Q., Tang X.Q. (2017). Coke formation on the surface of Ni/HZSM-5 and Ni–Cu/HZSM-5 catalysts during bio-oil hydrodeoxygenation. Fuel.

[27] Jahromi H., Agblevor F.A. (2018). Hydrodeoxygenation of Aqueous-Phase Catalytic Pyrolysis Oil to Liquid Hydrocarbons Using Multifunctional Nickel Catalyst. Ind. Eng. Chem. Res..

[28] Schmitt C.C., Reolon M.B.G., Zimmermann M., Raffelt K., Grunwaldt J.D., Dahmen N. (2018). Synthesis and Regeneration of Nickel-Based Catalysts for Hydrodeoxygenation of Beech Wood Fast Pyrolysis Bio-Oil. Catalysts.

[29] Alonso D.M., Wettstein S.G., Dumesic J.A. (2012). Bimetallic catalysts for upgrading of biomass to fuels and chemicals. Chem. Soc. Rev..

[30] Dongil A.B., Bachiller-Baeza B., Rodríguez-Ramos I., Fierro J.L.G., Escalona N. (2016). The effect of Cu loading on Ni/carbon nanotubes catalysts for hydrodeoxygenation of guaiacol. RSC Adv..

[31] Liu X., An W., Wang Y., Turner C.H., Resasco D.E. (2018). Hydrodeoxygenation of guaiacol over bimetallic Fe-alloyed (Ni, Pt) surfaces: Reaction mechanism, transition-state scaling relations and descriptor for predicting C–O bond scission reactivity. Catal. Sci. Technol..

[32] Han Q., Rehman M.U., Wang J., Rykov A., Gutiérrez O.Y., Zhao Y., Wang S., Ma X., Lercher J.A. (2019). The synergistic effect between Ni sites and Ni–Fe alloy sites on hydrodeoxygenation of lignin-derived phenols. Appl. Catal. B.

[33] Luo Z., Zheng Z., Li L., Cui Y.-T., Zhao C. (2017). Bimetallic Ru–Ni Catalyzed Aqueous-Phase Guaiacol Hydrogenolysis at Low H_2_ Pressures. ACS Catal..

[34] Huynh T.M., Armbruster U., Pohl M.-M., Schneider M., Radnik J., Hoang D.-L., Phan B.M.Q., Nguyen D.A., Martin A. (2014). Hydrodeoxygenation of Phenol as a Model Compound for Bio-oil on Non-noble Bimetallic Nickel-based Catalysts. ChemCatChem.

[35] Tran N.T.T., Uemura Y., Chowdhury S., Ramli A. (2016). Vapor-phase hydrodeoxygenation of guaiacol on Al-MCM-41 supported Ni and Co catalysts. Appl. Catal. A.

[36] Zhou M., Ye J., Liu P., Xu J., Jiang J. (2017). Water-Assisted Selective Hydrodeoxygenation of Guaiacol to Cyclohexanol over Supported Ni and Co Bimetallic Catalysts. ACS Sustain. Chem. Eng..

[37] Zanuttini M.S., Dalla Costa B.O., Querini C.A., Peralta M.A. (2014). Hydrodeoxygenation of m-cresol with Pt supported over mild acid materials. Appl. Catal. A.

[38] Zhu X., Lobban L.L., Mallinson R.G., Resasco D.E. (2011). Bifunctional transalkylation and hydrodeoxygenation of anisole over a Pt/HBeta catalyst. J. Catal..

[39] Brunauer S., Emmett P.H., Teller E. (1938). Adsorption of Gases in Multimolecular Layers. J. Am. Chem. Soc..

[40] Ghampson I.T., Pecchi G., Fierro J.L.G., Videla A., Escalona N. (2017). Catalytic hydrodeoxygenation of anisole over Re–MoO_x_/TiO_2_ and Re–VO_x_/TiO_2_ catalysts. Appl. Catal. B.

[41] Huynh T., Armbruster U., Kreyenschulte C., Nguyen L., Phan B., Nguyen D., Martin A. (2016). Understanding the Performance and Stability of Supported Ni–Co-Based Catalysts in Phenol HDO. Catalysts.

[42] Takanabe K., Nagaoka K., Nariai K., Aika K.-I. (2005). Titania-supported cobalt and nickel bimetallic catalysts for carbon dioxide reforming of methane. J. Catal..

[43] Reuel R.C., Bartholomew C.H. (1984). The stoichiometries of H_2_ and CO adsorptions on cobalt: Effects of support and preparation. J. Catal..

[44] Dongil A.B., Pastor-Pérez L., Escalona N., Sepúlveda-Escribano A. (2016). Carbon nanotube-supported Ni–CeO_2_ catalysts. Effect of the support on the catalytic performance in the low-temperature WGS reaction. Carbon.

[45] Ma Q., Wang D., Wu M., Zhao T., Yoneyama Y., Tsubaki N. (2013). Effect of catalytic site position: Nickel nanocatalyst selectively loaded inside or outside carbon nanotubes for methane dry reforming. Fuel.

[46] van Deelen T.W., Su H., Sommerdijk N.A.J.M., de Jong K.P. (2018). Assembly and activation of supported cobalt nanocrystal catalysts for the Fischer–Tropsch synthesis. Chem. Commun..

[47] van Deelen T.W., Yoshida H., Oord R., Zečević J., Weckhuysen B.M., de Jong K.P. (2020). Cobalt nanocrystals on carbon nanotubes in the Fischer-Tropsch synthesis: Impact of support oxidation. Appl. Catal. A.

[48] Dongil A.B., Ghampson I.T., García R., Fierro J.L.G., Escalona N. (2016). Hydrodeoxygenation of guaiacol over Ni/carbon catalysts: Effect of the support and Ni loading. RSC Adv..

[49] Han G.-H., Lee M.W., Park S., Kim H.J., Ahn J.-P., Seo M.-G., Lee K.-Y. (2019). Revealing the factors determining the selectivity of guaiacol HDO reaction pathways using ZrP-supported Co and Ni catalysts. J. Catal..

[50] Liu M., Zhang J., Zheng L., Fan G., Yang L., Li F. (2020). Significant Promotion of Surface Oxygen Vacancies on Bimetallic CoNi Nanocatalysts for Hydrodeoxygenation of Biomass-derived Vanillin to Produce Methylcyclohexanol. ACS Sustain. Chem. Eng..

[51] Mochizuki T., Chen S.-Y., Toba M., Yoshimura Y. (2014). Deoxygenation of guaiacol and woody tar over reduced catalysts. Appl. Catal. B.

[52] Herrera C., Ghampson I.T., Cruces K., Sepúlveda C., Barrientos L., Laurenti D., Geantet C., Serpell R., Contreras D., Melin V. (2020). Valorization of biomass derivatives through the conversion of phenol over silica-supported Mo-Re oxide catalysts. Fuel.

[53] Lu M., Sun Y., Zhang P., Zhu J., Li M., Shan Y., Shen J., Song C. (2019). Hydrodeoxygenation of Guaiacol Catalyzed by High-Loading Ni Catalysts Supported on SiO_2_–TiO_2_ Binary Oxides. Ind. Eng. Chem. Res..

[54] Dongil A.B., Pastor-Pérez L., Sepúlveda-Escribano A., García R., Escalona N. (2016). Hydrodeoxygenation of guaiacol: Tuning the selectivity to cyclohexene by introducing Ni nanoparticles inside carbon nanotubes. Fuel.

[55] Rather S.U. (2020). Preparation, characterization and hydrogen storage studies of carbon nanotubes and their composites: A review. Int. J. Hydrog. Energy.

[56] Dee la Peña O’Shea V.A., De la Piscina P.R., Homs N., Aromí G., Fierro J.L.G. (2009). Development of Hexagonal Closed-Packed Cobalt Nanoparticles Stable at High Temperature. Chem. Mater..

[57] Tsakoumis N.E., Patanou E., Lögdberg S., Johnsen R.E., Myrstad R., van Beek W., Rytter E., Blekkan E.A. (2019). Structure–Performance Relationships on Co-Based Fischer–Tropsch Synthesis Catalysts: The More Defect-Free, the Better. ACS Catal..

[58] Kitakami O., Sato H., Shimada Y., Sato F., Tanaka M. (1997). Size effect on the crystal phase of cobalt fine particles. Phys. Rev. B.

